# Supplementary material for: Implementation of oral health evidence-based practices in early care education settings across the U.S. during different COVID-19 periods
Source: PLoS One. 2025 Aug 18;20(8):e0323396. doi: 10.1371/journal.pone.0323396 (PMC12360538; doi:10.1371/journal.pone.0323396)
Supplement: Attachment — Submitted filename: Rebuttal Letter.docx [file pone.0323396.s003.docx]

**Manuscript PONE-D-25-17567**

July 1, 2025

**Rebuttal Letter**

Dear Nour Ammar,

Thank you for giving us the opportunity to submit a revised draft of the manuscript “*Implementation of oral health evidence-based practices in early care education settings across the U.S. during different COVID-19 periods*” for publication in the PLOS ONE Journal. We appreciate the time and effort that you and the reviewers dedicated to providing feedback on our manuscript and are grateful for the insightful comments and valuable improvements to our paper. We have incorporated all the suggestions made by the reviewers. These changes are highlighted using track changes in the file 'Revised Manuscript with Track Changes', and an unmarked version in the ‘Manuscript’ file.

Please find below a point-by-point response to the editor’s and reviewers’ comments and concerns. All page numbers refer to the 'Revised Manuscript with Track Changes' file.

**Comment 1.** Please ensure that your manuscript meets PLOS ONE's style requirements, including those for file naming.

**Response 1.** We revised the ‘title, author list, and affiliation’ page to align with the journal formatting guidelines.

**Comment 2.** We note that the grant information you provided in the ‘Funding Information’ and ‘Financial Disclosure’ sections do not match. When you resubmit, please ensure that you provide the correct grant numbers for the awards you received for your study in the ‘Funding Information’ section.

**Response 2.** We answered ‘no’ to receiving specific funding to do this work ‘Funding information’ and were unable to edit ‘Financial Disclosure’.

**Comment 3.** Please state what role the funders took in the study.

**Response 3.** First author, Dr. Tchoua, is supported by the T32 Cancer Health Disparities Training Grant (T32CA128582) from the National Cancer Institute of the National Institutes of Health. Service agreement contracts with state departments, non-profit, and academic institutions support Go NAPSACC's dissemination, allowing for the collection of data reported in this study. The funders had no role in study design, data collection and analysis, decision to publish, or preparation of the manuscript.

**Comment 4.** Data availability statement and data sharing plan.

**Response 4.** Due to contractual agreements with certain state partners, Go NAPSACC data cannot be made publicly available. However, de-identified or limited datasets may be made available upon reasonable request, contingent upon review and approval by the project team and compliance with institutional and contractual requirements. Contact: Go NAPSACC Initiative Team, gonapsacc@unc.edu.

**Reviewer #1:**

**Comment 1.** Abstract: The methods section is very brief. It does not mention that this is a cross-sectional study (which is an essential item according to the Strengthening the Reporting of Observational Studies in Epidemiology [STROBE] statement), nor does it specify that the data used span the period between July 2014 and February 2024. Additionally, the exclusion criteria were not described.

**Response 1.** Thank you for your comment. We revised the abstract to specify that the study is repeated cross-sectional, covers the period from July 2014 to February 2024, and includes the exclusion criteria. See Lines 26, 27-28, and 29-30.

**Comment 2.** Introduction: The introduction is well written; however, it lacks a description of the specific objectives of the study, including any pre-existing hypotheses.

**Response 2.** Thank you for your comment. This study is descriptive and does not have a pre-existing hypothesis. We clarified the objective as understanding the implementation of oral health practices in early care education settings before, during, and after the COVID-19 pandemic. See lines 104-106

**Comment 1.** Methods: Was any measure taken to reduce or control risk of bias? If so, please specify.

**Response 1.** Thank you for your comment. Yes. To minimize bias, (1) we defined exclusion criteria for data analysis (i.e., programs who did not participate in the Oral Health module), (2) our data was collected using a uniform self-assessment across all program participants, and (3) we acknowledged our limitations, self-reported data, and study design.

**Reviewer #2:**

**Comment 1.** Some references are either missing or are bit old.

**Response 1.** Thank you for your comment. We updated the manuscript with the most recent available references related to children’s oral health. Unfortunately, we are limited by currently available references.

**Comment 2.** Certain typographical errors

**Response 2.** We appreciate the reviewer bringing these to our attention. We have addressed typographical errors in the revised manuscript.

**Comment 3.** Some incomplete sentences or ambiguous

**Response 3.** Thank you for your comment. We carefully reviewed and corrected typographical errors throughout the manuscript.

**Manuscript Edits**

**Comment 1.** Introduction. “The COVID-19 pandemic disrupted many health practices across the US, including oral health practices in ECE settings. (reference).”

**Response 1.** We revised the sentence and added a reference 12. See lines 90-91.

**Comment 2.** Introduction. “These disruptions impacted not only the sustainability of programs but also the continuity of care and education for young children. The decline in oral health and the operational challenges faced by ECE programs are closely interconnected.(reference).”

**Response 2.** We added references 15 and 16 to support this statement. See lines 95-98.

**Comment 3.** Methods. “There are 11,431 ECE programs registered with Go NAPSACC across 23 states.(reference).”

**Response 3.** We added references 15 and 16 to support this statement. See lines 123-124.

Regards,

Phoebe Tchoua, DrPH, MPH
